# Urotensin II: A Key Role in Hormonal and Inflammatory Cytokine Regulation in COVID‐19 Patients

**DOI:** 10.1155/ijm/6760898

**Published:** 2026-10-22

**Authors:** Sazan Qadir Maulud

**Affiliations:** ^1^ Department of Biology College of Education Salahaddin University-Erbil Erbil Kurdistan Region Iraq su.edu.iq

**Keywords:** angiotensin II, COVID-19, endothelin-1, SARS-CoV-2, urotensin II

## Abstract

The COVID‐19 pandemic exhibits a wide spectrum of severities and pathophysiological mechanisms. The virus targets angiotensin‐converting enzyme 2 (ACE2) receptors, disrupting the renin–angiotensin–aldosterone system (RAAS) balance, which may explain various symptoms and risk factors. Fibrosis of the small airways is pivotal in the emergence of chronic obstructive pulmonary disease. Understanding these complex pathophysiological mechanisms is crucial for developing effective treatments and personalized approaches for COVID‐19 patients. The present study is aimed at analyzing the concentrations of the clinical and laboratory parameters—such as hematological, biochemical, serological, angiotensin II (ANG II), endothelin‐1 (ET‐1), urotensin II (UII), and cortisol parameters—in patients with SARS‐CoV‐2 and evaluating the elevation of these parameters related to the pathogenicity. The study included two distinct groups: a healthy control group (*n* = 25) and a COVID‐19 patient group (*n* = 75) during the period from August 2021 to January 2022. Cytokine levels were evaluated using commercially available enzyme‐linked immunosorbent assay (ELISA) kits. The comparative analysis revealed that severe cases of COVID‐19 have a significant elevation in the concentrations of hematological, biochemical, serological, and hormonal levels such as ANG II, ET‐1, UII, and cortisol, as well as a substantial decline in the concentration of LYM%, in comparison with mild cases of COVID‐19 and the control groups. Based on these findings, we concluded that ET‐1, ANG II, and UII may be associated with COVID‐19 pathophysiology and are linked to endothelial dysfunction, inflammation, and thrombosis.

## 1. Introduction

Severe acute respiratory syndrome coronavirus 2 (SARS‐CoV‐2), a pandemic coronavirus, was first detected in late 2019 [1]; it is a member of the Coronaviridae family and contains a single‐stranded, positive‐sense RNA (ssRNA) genome enclosed within a large lipid envelope [2]. The coronavirus genus *Beta* comprises viruses that are highly pathogenic, mainly novel SARS‐CoV‐2, Middle East respiratory syndrome coronavirus (MERS‐CoV), and severe acute respiratory syndrome coronavirus (SARS‐CoV) [3]. SARS‐CoV‐2 exhibits a high potential for human‐to‐human transmission; it is characterized by markedly high infectivity and an enhanced capacity to evade host immune responses, surpassing those observed in SARS‐CoV and MERS‐CoV [4]. COVID‐19 presents with a wide range of clinical symptoms, from mild manifestations such as fever, dry cough, shortness of breath, nasal inflammation, muscle aches, and fatigue, to more severe outcomes like pneumonia, RNAemia, cardiac injury, acute respiratory distress syndrome (ARDS), multiorgan dysfunction, and mortality [5]. Current research has also investigated conserved target sequences within the RNA genome of SARS‐CoV‐2 as possible targets for virus‐specific antiviral strategies [6].

Angiotensin‐converting enzyme 2 (ACE2) is bound by the spike (S) glycoprotein of SARS‐CoV‐2, thereby interfering with the homeostatic balance of the renin–angiotensin–aldosterone system (RAAS), which may account for the diverse clinical symptoms and associated risk factors observed in infected individuals [7]. This imbalance leads to increased angiotensin II (ANG II) activity and decreased angiotensin 1–7, potentially triggering inflammation, thrombosis, and other adverse reactions. There is accepted evidence that virus‐induced endothelium damage in progressively severe COVID‐19 illness may develop excessive vasoconstriction, inflammation, and thrombosis [4]. Severe indications of COVID‐19 are linked to the emergence of a cytokine storm [8]. Nonetheless, there have been recent findings suggesting that a vasoactive peptide storm, comprising bradykinin, substance P, and neurotensin, may also play a role in microvascular permeability and inflammation. Histological evaluation of tissue from COVID‐19 patients with acute lung, renal, and cardiac damage revealed the presence of viral inclusion and lymphocyte infiltration associated with apoptotic endothelium cells [9].

ACE2 is an important part of the renin–angiotensin system (RAS), whereby ANG II serves as the main peptide produced through the enzymatic activity of ACE and ACE2. This highlights the physiological significance of ANG II throughout evolutionary history [10]. ANG II induces inflammatory reactions through the activation of the angiotensin II Type 1 receptor (AT1R) that leads to a rise in secretion of inflammatory cytokines—in particular, tumor necrosis factor‐alpha (TNF‐*α*) and interleukin‐6 (IL‐6) [3]. ANG II′s functional effects are associated with the mechanism by which SARS‐CoV‐2 gains access into pulmonary cells, thus triggering the synthesis of proinflammatory cytokines, primarily IL‐6 and TNF‐*α*. Notably, IL‐6 is considered one of the most reliable biomarkers of fatalities during hospitalization [11]. Studies have shown that virus‐induced downregulation of ACE2 disrupts the homeostatic balance, favoring heightened ANG II activity, which contributes to inflammation, fibrosis, and oxidative stress in the lungs, heart, and kidneys [12]. Conversely, the reduction of ACE2 expression in COVID‐19 patients may lead to the accumulation of ANG II [13].

Moreover, endothelin‐1 (ET‐1), a powerful vasoconstrictor secreted by the endothelial cells that is increased in reaction to bacterial endotoxins and various proinflammatory cytokines, including TNF‐*α* and IL‐6, is central to the control of cardiovascular tone and homeostasis. In COVID‐19 patients, direct viral injury to endothelial cells has been associated with elevated ET‐1 expression [14]. Thereby, elevation of ET‐1 levels contributes to vasoconstriction, endothelial inflammation, and microvascular thrombosis key features of severe disease progression [15]. Moreover, urotensin II (UII) is identified as the most effective endogenous vasoconstrictor, playing a role in the development of dysfunctional endothelial cells, inflammation, and fibrosis. Elevated UII levels are frequently observed in cardiovascular diseases and may contribute to vascular complications associated with COVID‐19, such as pulmonary hypertension and endothelial damage [16, 17]. Recent investigations have shown that UII has significant vasoconstrictor action, with strength approximately 100 times greater than that of ET‐1 [18]. Cortisol, a steroid hormone, follows a regular diurnal rhythm under stress‐free conditions, characterized by a peak in the morning and lower levels in the evening. However, any form of stress, including stress of a pandemic, can induce significant stress responses, particularly in individuals with COVID‐19 [19, 20]. Elevated serum cortisol is a physiological response that activates both the immunological and cardiovascular systems to regulate stress, primarily through the hypothalamic–pituitary–adrenal (HPA) axis [21]. Currently, there is limited clinical information about the release of ANG II, ET‐1, and UII in COVID‐19 patients and their effect on the patho‐immunophysiological condition of these individuals. The study focused on role of ANG II, ET‐1, UII, and cortisol levels and its effective ways on some biochemical, hematologic, and immunologic predictive parameters related to COVID‐19 patients′ severity. Furthermore, understanding these complex pathophysiological pathways is important for improving the understanding of disease mechanisms and may inform future therapeutic research.

## 2. Materials and Methods

### 2.1. Patients and Samples

The emergency hospitals included Emarat and Rozhawa in Erbil, Iraq, which served as referral centers for COVID‐19 patients. Hospitalized patients who showed up with symptoms of the upper respiratory tract, such as nasal congestion, sneezing, sore throat, rhinorrhea, nasal discharge, and cough, were enrolled in this study. The patients were categorized as mild or severe COVID‐19 based on clinical presentation and oxygenation status on admission in line with institutional guidelines in favor of the WHO criteria. Mild cases were characterized by respiratory symptoms without hypoxia (SpO_2_ ≥ 94*%* on room air), whereas severe cases were defined by respiratory distress (SpO_2_ < 94*%*) or radiological evidence of pneumonia requiring supplemental oxygen.

The inclusion criteria included a minimum age of 18 years and reverse transcription‐polymerase chain reaction (RT‐PCR)–proven infection with SARS‐CoV‐2. Samples were taken upon hospital admission before specific pharmacological treatment, such as systemic corticosteroids, antiviral agents, and anticoagulants. Patients that had chronic endocrine disorders, autoimmune diseases, malignancy, and those that had previously been treated with systemic corticosteroids or RAS modulating drugs were excluded. Potential comorbidities were noted (major comorbidities: hypertension, diabetes mellitus, and cardiovascular disease).

The nasopharyngeal swab samples were collected in 75 patients and put in viral transport medium (VTM) (UTM‐RT, Copan, Italy). The samples were carried to the laboratory and kept at −80°C. A RT‐PCR diagnosed the SARS‐CoV‐2 at the Central Public Health Laboratory (CPHL), Erbil, Kurdistan Region, Iraq. Moreover, blood samples of COVID‐19 patients were taken to assess biochemical, hematological, immunological, and hormonal values.

### 2.2. Viral RNA Extraction and Real‐Time PCR

AccuPrep Viral RNA Extraction Kit (Bioneer‐Corporation, Korea) was used to extract RNA using nasopharyngeal samples according to the instructions of the manufacturer. The swab samples have been vortexed at high speed for a duration of 10 s, and RNA was extracted from 200 *μ*L of VTM (UTM‐RT), with an eluted volume of 50 *μ*L. The DIAGNOVITAL SARS‐CoV‐2 Real‐Time PCR Kit was utilized for detection of SARS‐CoV‐2 via the Rotor‐Gene Q Real‐Time PCR Detecting System (QIAGEN), following the manufacturer′s protocol.

### 2.3. Biochemical and Hematological Analysis

A completely automated three‐part differential hematological analyzer (Medonic *α*‐Alpha, Germany) was used to perform complete blood count (CBC) analysis, which included LYM%, GRA%, and MID%. The whole process was maintained according to the manufacturer′s recommendations. C‐reactive protein (CRP) levels in blood serum and D‐dimer levels in blood plasma were quantified using a kit with an autoanalyzer (Nano‐Checker 710 Reader, Nano‐Ditech, United States). The CRP levels were assayed by using a kit (Nano‐Check TM CRP kits from Nano‐Ditech, United States), and D‐dimer levels were assayed by using a kit (Nano‐Check TM D‐dimer kit from Nano‐Ditech, United States). All procedures were conducted following the manufacturer′s guidelines. Serum ferritin concentrations were measured using a commercially available kit on an automated analyzer (cobas e 411, Hitachi). The ferritin levels were assayed by using a kit (Elecsys Ferritin kits from Cobas, Germany). The whole process was maintained following to the manufacturer′s recommendations.

### 2.4. Serology Test

Blood specimens were taken to extract serum from COVID‐19 patients, which was then aliquoted and preserved at −20°C until analysis. The concentrations of IFN‐*γ*, TNF‐*α*, and IL‐6 were evaluated using publicly accessible enzyme‐linked immunosorbent assay (ELISA) kits, following the manufacturer′s guidelines. Serum levels of TNF‐*α* and IFN‐*γ* in the samples were measured using human (TNF‐*α*) ELISA and human (IFN‐*γ*) ELISA kits (Sunredbio, China), respectively. The samples′ serum level of IL‐6 was measured using the human interleukin ELISA kit (Demeditec, Germany) at 450 nm, as directed by the manufacturer.

### 2.5. Hormonal Test

For ET‐1, UII, ANG II, and cortisol, serum levels in the samples were measured by using human (ET‐1) ELISA, human (UII) ELISA, human (ANG II) ELISA, and human (cortisol) ELISA kits (Sunredbio, China) The optical density (OD) was evaluated at 450 nm following the manufacturer′s guidelines.

### 2.6. Statistical Analysis

The statistical analyses were given with the help of GraphPad Prism (Version 8.0, GraphPad Software Inc., San Diego, California, United States). The statistics are presented in means and standard deviation (SD). The control, mild, and severe COVID‐19 groups were compared by means of one‐way analysis of variance (ANOVA) with post hoc multiple‐comparison test of Tukey. Variables were correlated using Pearson correlation coefficient. The *p* value of less than 0.05 was considered to be statistically significant.

Data distribution was also determined before the application of parametric tests based on Shapiro–Wilk test and homogeneity of variances based on Levene test. Parametric methods were used to analyze variables that met these assumptions as above.

## 3. Results

The results of the current study, which included 75 hospitalized patients diagnosed with COVID‐19 positive (+ve) via PCR tests were selected between August 2021 and January 2022. Descriptive data for the study population are presented in Table 1, which shows that the percentage of COVID‐19 infection was higher in men (56%) than in women (44%). There were 25 control groups, 29 mild cases, and 46 severe cases, respectively. Body mass was higher in severe COVID‐19 cases than in mild cases, as shown in Figure 1.

**Table 1:** Baseline demographics of SARS‐CoV2 positive patients.

Characteristic	Control (*n* = 25)	Mild COVID‐19 (*n* = 29)	Severe COVID‐19 (*n* = 46)
Sex, *n* (%)	—	42 (56%)	33 (44%)
Age (years), mean ± SD	—	58.66 ± 14.05	56.11 ± 16.39
Body mass (kg), mean ± SD	—	82.28 ± 13.07	89.41 ± 12.84

**Figure 1:**
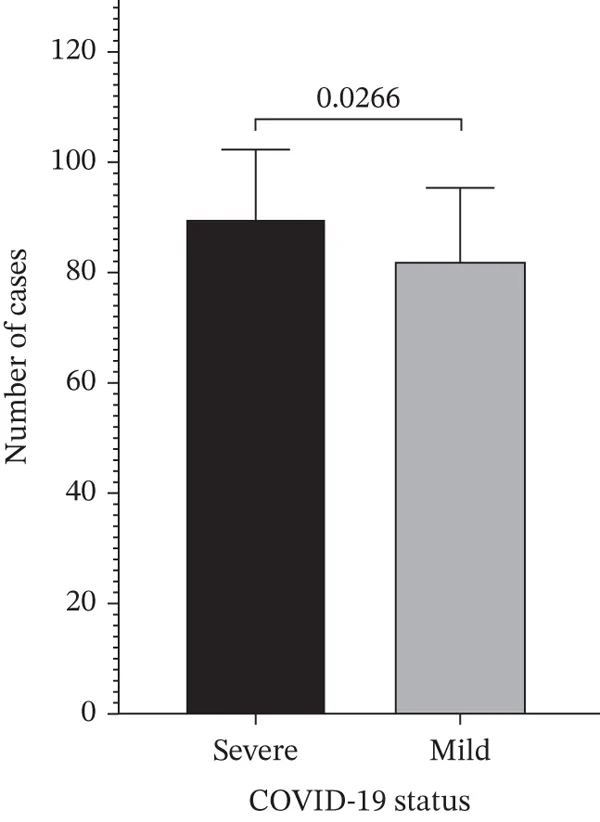
The body mass of SARS‐CoV‐2 positive patients. The data are expressed as mean ± SD values for each group.

Hematological parameters varied according to disease severity. MID% was considerably higher in severe COVID‐19 patients (19.28 ± 21.35) than in mild cases (7.52 ± 1.92) and controls (6.89 ± 2.32). No considerable difference was found between mild cases and controls (Figure 2A). Similarly, GRA% was significantly increased in severe cases (92.30 ± 2.07) compared with mild cases (83.29 ± 2.99) and controls (59.94 ± 9.57) (Figure 2B), whereas LYM% was markedly decreased in severe cases (4.95 ± 2.09) compared with mild cases (19.33 ± 7.87) and controls (33.17 ± 7.47) (Figure 2C).

**Figure 2:**
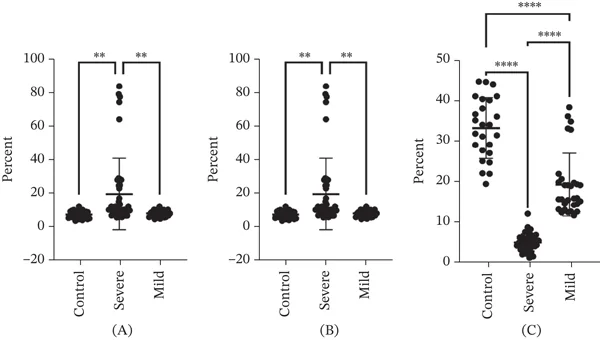
Hematological parameters according to COVID‐19 severity. (A) MID%, (B) GRA%, and (C) LYM% levels in control, mild COVID‐19, and severe COVID‐19 groups. Data are expressed as mean ± SD. Statistical significance was determined using one‐way ANOVA with Tukey′s post hoc test. ∗*p* < 0.05, ∗∗*p* < 0.01, ∗∗∗*p* < 0.001, and ∗∗∗∗*p* < 0.0001.

The biochemical parameters, including CRP, D‐dimer, and ferritin, were significantly associated with the severity of SARS‐CoV‐2 infection (Figure 3A–C). In severe COVID‐19 patients, the mean levels of CRP, D‐dimer, and ferritin were markedly elevated, reaching 20.07 ± 5.74 mg/L, 3114.38 ± 1130.27 ng/mL, and 1629.78 ± 414.97 ng/mL, respectively. In contrast, mild cases showed lower values of 6.37 ± 2.52 mg/L, 855.009 ± 215.06 ng/mL, and 358.37 ± 158.31 ng/mL, respectively, whereas the control group exhibited the lowest levels (1.68 ± 0.61 mg/L, 167.96 ± 71.42 ng/mL, and 119.48 ± 43.11 ng/mL, respectively).

**Figure 3:**
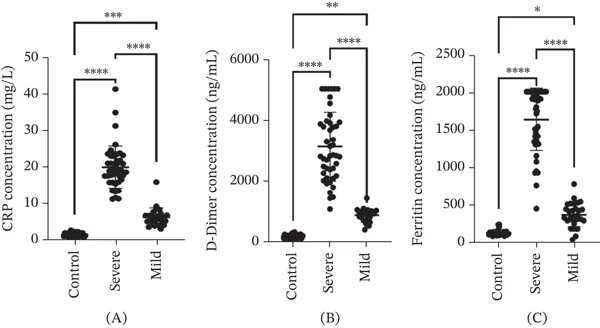
Inflammatory biomarker levels according to COVID‐19 disease severity. (A) CRP concentrations, (B) D‐dimer concentrations, and (C) ferritin concentrations in control, mild COVID‐19, and severe COVID‐19 groups. Data are expressed as mean ± SD. Statistical significance was determined using one‐way ANOVA followed by Tukey′s post hoc test. ∗*p* < 0.05, ∗∗*p* < 0.01, ∗∗∗*p* < 0.001, and ∗∗∗∗*p* < 0.0001.

The serum cytokine levels, including IL‐6, IFN‐*γ*, and TNF‐*α*, were evaluated across different groups of COVID‐19 patients (Figure 4A–C). IL‐6 concentrations were significantly higher in severe cases (535.15 ± 272.54 pg/mL) compared with mild cases (243.63 ± 130.44 pg/mL) and the control group (52.1 ± 8.28 pg/mL) (Figure 4A). Similarly, IFN‐*γ* levels were significantly higher in severe cases (110.67 ± 41.01 ng/L) compared with mild cases (90.97 ± 19.98 ng/L) and the control group (50.91 ± 19.37 ng/L) (Figure 4B). TNF‐*α* concentrations also showed a marked increase in severe cases (143.67 ± 77.91 ng/L) compared with mild cases (90.81 ± 17.21 ng/L) and the control group (78.93 ± 9.69 ng/L) (Figure 4C). Overall, all cytokines were significantly elevated in severe COVID‐19 patients compared with mild cases and controls.

**Figure 4:**
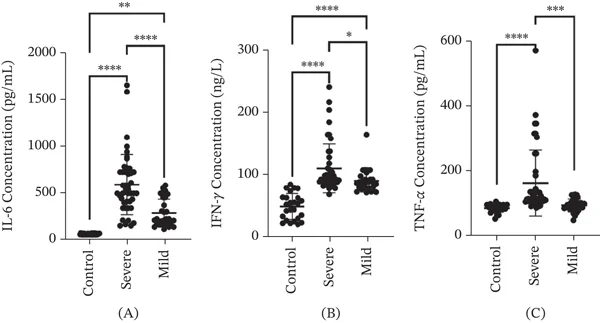
Cytokine levels according to COVID‐19 disease severity. (A) IL‐6 concentrations, (B) IFN‐*γ* concentrations, and (C) TNF‐*α* concentrations in control, mild COVID‐19, and severe COVID‐19 groups. Data are expressed as mean ± SD. Statistical significance was determined using one‐way ANOVA followed by Tukey′s post hoc test. ∗*p* < 0.05, ∗∗*p* < 0.01, ∗∗∗*p* < 0.001, and ∗∗∗∗*p* < 0.0001.

Serum concentrations of hormonal markers are presented in Figure 5A–D. Hormonal tests, including UII, ET‐1, ANG II, and cortisol, were evaluated in different groups of COVID‐19 patients. The study revealed that these hormone levels were increased in COVID‐19 patients in comparison with those observed for the control group in both males and females. In males, UII levels were substantially increased in severe cases (3.49 ± 3.006 pmol/L) compared with mild cases (1.92 ± 0.74 pmol/L) and the control groups (1.2 ± 0.22 pmol/L). Additionally, the mild cases and the control group did not show any significant differences. Nevertheless, no significant differences in UII levels were observed among female groups. In males ET‐1 (ng/L) concentrations were substantially increased in severe cases (76.23 ± 57.603 ng/L) compared with both mild cases (30.04 ± 11.98 ng/L) and control group (8.29 ± 1.63 ng/L), whereas in females, ET‐1 levels were considerably increased in severe cases (73.89 ± 55.91 ng/L) compared with the control group (8.95 ± 1.52 ng/L), and slightly elevated compared with mild cases (43.95 ± 18.88 ng/L), and no significant differences were observed between mild cases and controls. Moreover, there is a substantial difference among severe cases, mild cases, and control groups in the level of ANG II. The ANG II levels in both males and females were substantially increased in severe cases (787.77 ± 395.22 and 776.703 ± 297.51 pg/mL) compared with both mild cases (403.09 ± 138.302 and 417.602 ± 125.89 pg/mL) and the control group (72.34 ± 19.86 and 70.57 ± 13.56 pg/mL), respectively. In the same context, we found the cortisol levels in both males and females were markedly elevated in severe cases (476.39 ± 283.37 and 456.37 ± 317.24 nmol/L) compared with both mild cases (257.95 ± 69.17 and 292.69 ± 48.005 nmol/L) and control groups (124.79 ± 13.82 and 126.12 ± 10.97 nmol/L), respectively, but there are no significant differences among mild cases and the control group.

**Figure 5:**
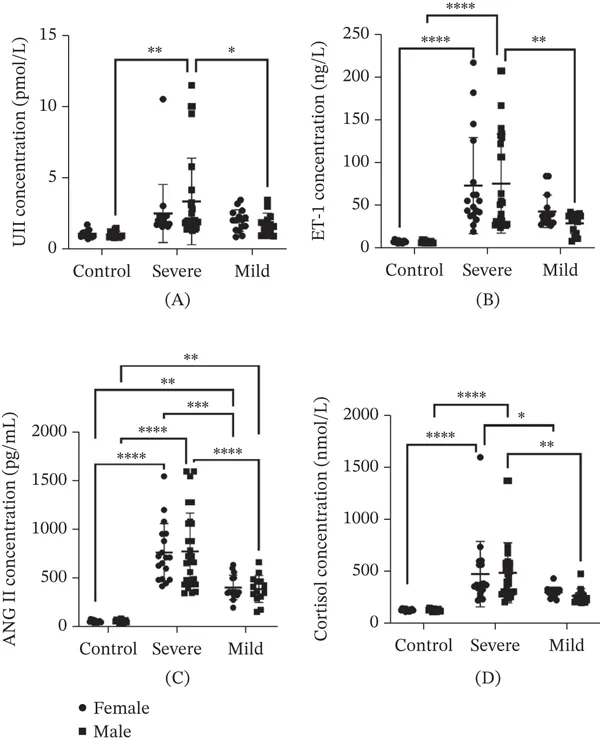
Hormonal biomarker levels according to COVID‐19 disease severity and sex distribution. (A) UII concentrations, (B) ET‐1 concentrations, (C) ANG II concentrations, and (D) cortisol concentrations in control, mild COVID‐19, and severe COVID‐19 groups. Circles represent females, and squares represent males. Data are expressed as mean ± SD. Statistical significance was determined using one‐way ANOVA followed by Tukey′s post hoc test. ∗*p* < 0.05, ∗∗*p* < 0.01, ∗∗∗*p* < 0.001, and ∗∗∗∗*p* < 0.0001.

Pearson correlation analysis in COVID‐19 patients showed significant associations between hormonal and inflammatory parameters. UII had a weak positive correlation with GRA% (*r* = 0.20, *p* = 0.088) and borderline negative one with LYM% (*r* = −0.23, *p* = 0.051) (Figure 6A). In contrast, ET‐1 showed a moderate positive correlation with GRA% (*r* = 0.42, *p* < 0.001), and a negative correlation with LYM% (*r* = −0.29, *p* = 0.014) (Figure 6B). Similarly, ANG II exhibited a significant positive correlation with GRA% (*r* = 0.47, *p* < 0.001) and a negative correlation with LYM% (*r* = −0.48, *p* = 0.001) (Figure 6C). These findings indicate a consistent relationship between elevated vasoactive hormones and inflammatory hematological markers in COVID‐19 patients.

**Figure 6:**
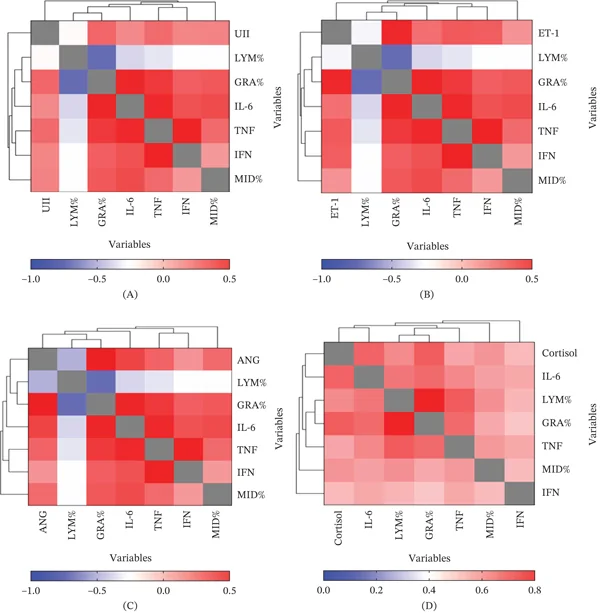
Heatmap analysis of Spearman correlation coefficients between hormonal biomarkers and hematological/inflammatory parameters in COVID‐19 patients. (A) UII, (B) ET‐1, (C) ANG II, and (D) cortisol correlation profiles with hematological and biochemical markers. Red indicates positive correlations, whereas blue indicates negative correlations, with color intensity corresponding to correlation strength.

The correlations among hormonal, inflammatory, and biochemical parameters are presented in Figure 7A–D. ANG II levels showed positive correlations with CRP (*r* = 0.48, *p* < 0.001), D‐dimer (*r* = 0.44, *p* < 0.001), and ferritin (*r* = 0.45, *p* < 0.001) (Figure 7C). Moreover, there was a statistically significant positive correlation between cortisol levels and IL‐6 (*r* = 0.50, *p* < 0.001), as well as a negative correlation between cortisol levels and LYM% (*r* = −0.31, *p* = 0.007) (Figure 7D). These findings indicate that vasoactive‐ and stress‐related hormonal pathways are closely associated with inflammatory and hematological changes in COVID‐19 patients (*n* = 75).

**Figure 7:**
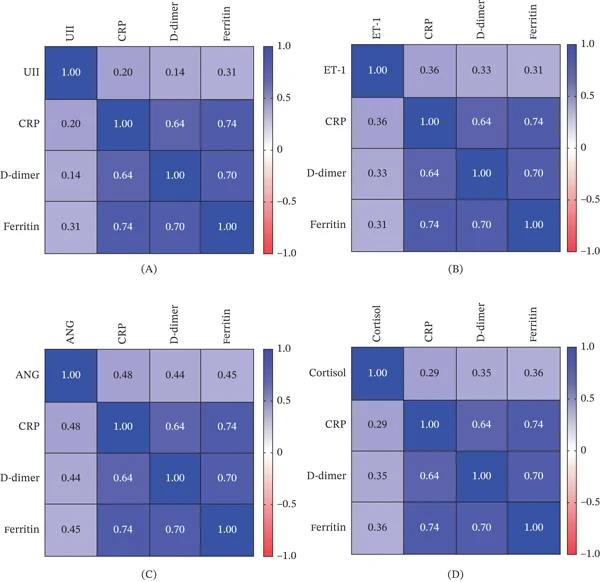
Correlation heatmaps illustrating the Spearman correlation coefficients between hormonal biomarkers and serological inflammatory markers in COVID‐19 patients. (A) UII, (B) ET‐1, (C) ANG II, and (D) cortisol correlations with CRP, D‐dimer, and ferritin levels. Blue and red color gradients indicate the strength and direction of correlations, whereas numerical values within each cell represent Spearman correlation coefficients (*r*).

The overall correlation pattern among UII, ET‐1, ANG II, and cortisol is presented in Figure 8. The analysis revealed a consistent positive association between these hormonal markers, indicating coordinated activation of vasoactive‐ and stress‐related pathways in COVID‐19 patients. In particular, UII levels showed positive relationships with ANG II and cortisol, whereas ET‐1 was also positively correlated with both UII and cortisol. Similarly, ANG II demonstrated strong positive associations with cortisol levels. Overall, these findings suggest that these hormonal systems are closely interconnected and may act synergistically in response to disease severity.

**Figure 8:**
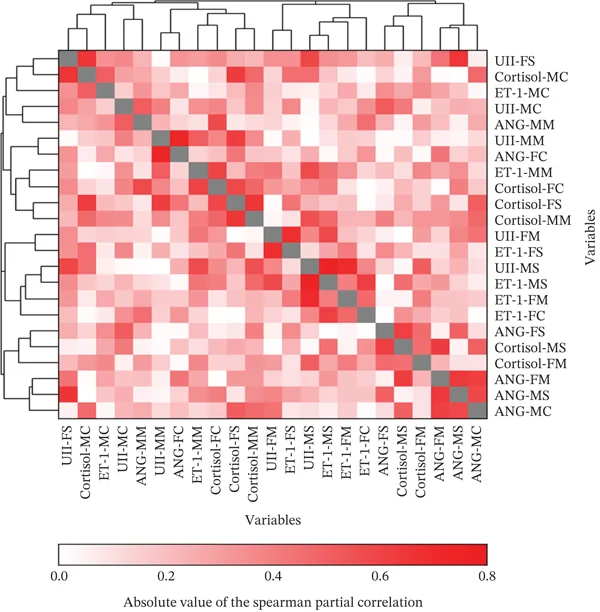
Heatmap showing the overall correlation between UII, ET‐1, ANG II, and cortisol.

## 4. Discussion

Currently, COVID‐19 has been having a great impact on more than 210 countries and regions around the world [22]. The demographic and the clinical features of COVID‐19 were extensively documented [23, 24]. Nevertheless, the biological processes that underlie the pathogenesis of COVID‐19 remain to be completely identified. This study evaluates the concentrations of several inflammatory parameters, such as hematological, biochemical, immunological biomarkers, and hormones then used as comparable factors among mild to severe manifestations of SARS‐CoV‐2 and finally with controls.

Clinical research has demonstrated that significant cases are associated with elevated plasma inflammatory mediators along with inflammation of the vascular system. In COVID‐19 cases, serum levels of lymphocytes and proinflammatory cytokines such as IFN‐*γ*, TNF‐*α*, IL‐6, CRP, ferritin, and D‐dimer are inflamed [23, 25, 26]. In the present research, we determined current literature‐based prevalent hematological markers that are employed to evaluate clinical severity and progression of the illness. The levels of LYM%, GRA%, and MID% have been shown to correlate with the severity of COVID‐19 and were identified as possible predictors of patient clinical outcomes [27]. Both SARS‐CoV and MERS‐CoV have been revealed to induce lymphopenia, which is linked to the activation of apoptotic processes both the intrinsic and extrinsic processes [28]. We assessed plasma CRP levels, which serve as a global indicator of liver‐induced inflammation [28]. A retrospective investigation by Para et al. [29] revealed that hospitalized COVID‐19 cases with increased ferritin levels faced a significantly higher likelihood of death. Furthermore, blood coagulation parameters in SARS‐CoV‐2‐positive individuals seem to be substantially changed in comparison to control groups. Notably, D‐dimer levels were found to be markedly elevated [30]. Additionally, the severity of the diseases was significantly correlated with the concentration of IFN‐*γ*, TNF‐*α*, and IL‐6 [31]. The acute COVID‐19 disease is frequently accompanied by an increased inflammatory response, which can result in a fatal cytokine storm in severe disease, frequently resulting in multiorgan failure and mortality [32]. Consistent with previous research, our findings demonstrated that the levels of biochemical and inflammatory markers such as CRP, D‐dimer, ferritin, IFN‐*γ*, TNF‐*α*, and IL‐6 were significantly altered in severe manifestations of COVID‐19 disease, as contrasted with mild cases and control groups.

In individuals suffering from COVID‐19, several predictive indicators and proinflammatory cytokines from various mechanisms have been examined. Nonetheless, to the best of our knowledge, a lack of thorough data regarding endothelium signals that are ANG II, ET‐1, and UII on COVID‐19 patients has not been thoroughly studied yet. These markers likely contribute to the pathogenic host response and may exacerbate disease severity in COVID‐19 [33]. SARS‐CoV‐2 enhances ANG II synthesis by attaching to ACE2 upon entry into cells. This activates the body′s innate defensive mechanisms. Additionally, it eliminates ACE2, which means that inflammation stimulated by ANG II remains persistent [13]. ANG II′s function in the regulation of cell signaling in immune‐related illnesses and inflammation is suggested by its ability to stimulate cell proliferation and the generation of cytokines [34]. It is evident that the impacts of the infection with SARS‐CoV‐2 will be insignificant when the viral load is down. However, in instances in which the viral load is elevated and the level of ACE2 receptors is likely to rise, as SARS‐CoV‐2 disseminates more extensively within tissues, the activation of the ANG II pathway is intensified, causing an exacerbated inflammatory response [9, 10]. Our results, which agree with the previous research, indicate that severe COVID‐19 disease results are more prevalent at elevated serum ANG II levels. ET‐1 is an effective vasoconstrictor of human blood‐vessels that is highly expressed and synthesized by endothelial cells. ET‐1 is regarded as a promising target for therapeutic intervention due to its critical function in vascular homeostasis and pathophysiology [35]. ET‐1 is generated in responses to extracellular stimulus, particularly inflammatory cytokines [14]. A persuasive rationale for investigating ET‐1 in COVID‐19 patients is provided by the findings of previous investigations on other types of diseases [14, 15]. Further investigations by Willems et al. [36] and Sayed et al. [37] reported elevated ET‐1 levels in specific cohorts of 36 COVID‐19–infected patients throughout the acute stage of the disease in comparison with the previously established noninfected control group. Our findings are consistent with previous studies demonstrating a sustained, virus‐induced elevation in endothelial peptide levels, particularly ET‐1, throughout the acute stage of COVID‐19 disease. This increase has been correlated with the clinical severity of the infection.

The biological effects of UII are mediated through the activation of its G‐protein–coupled urotensin receptor (UT) to cause intracellular signals such as phospholipase C, protein kinase C, mitogen‐activated protein kinase (MAPK), and calcium‐dependent cascades. Postsynaptic mechanisms stimulated by these promote vasoconstriction, endothelial stimulation, oxidative stress, and proinflammatory gene expression [16, 17]. The fact that some of these signaling pathways overlap with the pathways activated during SARS‐CoV‐2 infection is particularly true of the pathways that mediate endothelial dysfunction and cytokine [13, 34]. Nevertheless, UII indicators are signaled by activating UT receptors and triggering calcium and MAPK signals, whereas SARS‐CoV‐2 pathogenesis is mediated mostly by ACE2 downregulation, renin–angiotensin disequilibrium, and innate immune responses [13]. These mechanisms can interact to increase the vascular and inflammatory damage in COVID‐19. As such, a high UII in the current research could indicate the presence of vasoactive stress signaling that combines with SARS‐CoV‐2–induced endothelial and inflammatory activities.

The current results indicate the presence of strong correlations among high levels of ET‐1, ANG II, UII, and cortisol with inflammatory and hematological changes in patients with COVID‐19. The relationships indicate that the severity of a disease and systemic inflammation correlates with the activation of vasoactive‐ and stress‐related hormonal mechanisms, which are described in the literature as the participation of vasoactive peptides and stress hormones in the process of inflammatory reactions and endothelial responses to COVID‐19 [16, 38]. The age, the body mass index, and comorbid diseases, such as hypertension, diabetes mellitus, and cardiovascular disease, are known to influence vasoactive and hormonal processes, such as RAAS activation, endothelin signaling, and cortisol regulation. Even though the baseline demographic traits were recorded (Table 1), it is not possible to completely control them in the present study as the study design is observational. Moreover, the present study has analyzed several biomarkers, which can predispose to occurrence of Type I error, that is, the statistical comparison occurs more than once. Though conventional statistical significance levels were used in determining statistical significance, there was no formal correction on multiple testing since it was an exploratory study. Thus, such associations must be taken with reasonable care.

Even though these observations indicate a possible role of endothelial and renin–angiotensin pathways in the pathophysiology of COVID‐19, the cross‐sectional nature of the observations in this study precludes the possibility of causal relationships. These pathways are further subject to mechanistic and interventional studies in order to demystify whether their modulation has therapeutic significance.

## 5. Conclusion

In conclusion, elevated levels of ET‐1, ANG II, UII, and cortisol were strongly associated with inflammatory, hematological, and biochemical markers of disease severity in COVID‐19 patients. These findings highlight the coordinated involvement of vasoactive‐ and stress‐related hormonal pathways in disease pathophysiology. However, due to the observational nature of the study, causal relationships cannot be established, and further longitudinal and mechanistic studies are required to evaluate their potential as therapeutic targets.

## Funding

No funding was received for this manuscript.

## Conflicts of Interest

The author declares no conflicts of interest.

## Data Availability

The data that support the findings of this study are available in the Supporting Information of this article.
